# Quality of Web-Based Educational Interventions for Clinicians on Human Papillomavirus Vaccine: Content and Usability Assessment

**DOI:** 10.2196/cancer.9114

**Published:** 2018-02-16

**Authors:** Brittany L Rosen, James M Bishop, Skye L McDonald, Jessica A Kahn, Gary L Kreps

**Affiliations:** ^1^ School of Human Services University of Cincinnati Cincinnati, OH United States; ^2^ Department of Pediatrics Cincinnati Children's Hospital Medical Center Cincinnati, OH United States; ^3^ College of Medicine University of Cincinnati Cincinnati, OH United States; ^4^ Center for Health and Risk Communication Department of Communication George Mason University Fairfax, VA United States

**Keywords:** papillomavirus vaccines, internet, program evaluation, health personnel

## Abstract

**Background:**

Human papillomavirus (HPV) vaccination rates fall far short of Healthy People 2020 objectives. A leading reason is that clinicians do not recommend the vaccine consistently and strongly to girls and boys in the age group recommended for vaccination. Although Web-based HPV vaccine educational interventions for clinicians have been created to promote vaccination recommendations, rigorous evaluations of these interventions have not been conducted. Such evaluations are important to maximize the efficacy of educational interventions in promoting clinician recommendations for HPV vaccination.

**Objective:**

The objectives of our study were (1) to expand previous research by systematically identifying HPV vaccine Web-based educational interventions developed for clinicians and (2) to evaluate the quality of these Web-based educational interventions as defined by access, content, design, user evaluation, interactivity, and use of theory or models to create the interventions.

**Methods:**

Current HPV vaccine Web-based educational interventions were identified from general search engines (ie, Google), continuing medical education search engines, health department websites, and professional organization websites. Web-based educational interventions were included if they were created for clinicians (defined as individuals qualified to deliver health care services, such as physicians, clinical nurses, and school nurses, to patients aged 9 to 26 years), delivered information about the HPV vaccine and how to increase vaccination rates, and provided continuing education credits. The interventions’ content and usability were analyzed using 6 key indicators: access, content, design, evaluation, interactivity, and use of theory or models.

**Results:**

A total of 21 interventions were identified, out of which 7 (33%) were webinars, 7 (33%) were videos or lectures, and 7 (33%) were other (eg, text articles, website modules). Of the 21 interventions, 17 (81%) identified the purpose of the intervention, 12 (57%) provided the date that the information had been updated (7 of these were updated within the last 6 months), 14 (67%) provided the participants with the opportunity to provide feedback on the intervention, and 5 (24%) provided an interactive component. None of the educational interventions explicitly stated that a theory or model was used to develop the intervention.

**Conclusions:**

This analysis demonstrates that a substantial proportion of Web-based HPV vaccine educational interventions has not been developed using established health education and design principles. Interventions designed using these principles may increase strong and consistent HPV vaccination recommendations by clinicians.

## Introduction

The human papillomavirus (HPV) causes almost all cervical cancers, 50% of vulvar cancers, 65% of vaginal cancers, 90% of anal cancers, and 35% of penile cancers [1]. Recent studies have shown that the incidence of oral cancers caused by HPV is increasing [2-4]. The 9-valent HPV vaccine (9vHPV) is nearly 100% effective in preventing precancerous lesions caused by 7 genotypes [5], accounting for 81% of cervical cancer cases [6]. Despite ample evidence that licensed HPV vaccines are safe to use and effective in preventing certain anogenital cancers, only 42% of adolescent females and 28% of adolescent males have completed the HPV vaccine series [7]. These HPV vaccination rates fall short of Healthy People 2020’s objective of 80% coverage for girls and boys aged between 13 and 15 years [8].

Missed opportunities for clinicians to recommend and administer the vaccine, as well as a lack of strong and consistent recommendations by clinicians who do recommend the vaccine, are primary reasons for low HPV vaccination rates in the United States [9]. Factors contributing to missed clinical recommendation opportunities and insufficiently strong and consistent recommendations include providers’ limited knowledge of HPV and the vaccine, discomfort discussing a topic related to sexual behavior, concerns about vaccine safety, parental resistance, preference for vaccinating older adolescents, and lack of time or incentive to educate parents about the vaccine [10-19]. While improving communication between health care providers and parents is considered a critical component to increasing vaccination rates [20-26], health care providers report they do not feel well-prepared to provide strong vaccination recommendations [15,27,28]. Additionally, 75% of clinicians expressed they would benefit from continuing education about recommending the HPV vaccine [29].

To improve clinicians’ HPV vaccine recommendations, numerous Web-based HPV vaccine educational interventions for clinicians have been created. Web-based educational interventions have become a popular delivery method for health care professionals to obtain continuing education (CE) [30], as Web-based interventions provide an opportunity to quickly update and address health education topics at low cost [31]. With rapid proliferation of Web-based health education programs, there is a potential risk of neglecting fundamental health communication and education design principles in these programs that are important to ensure their efficacy [32-34].

Web-based CE interventions created using strategic health communication design principles—communicating effectively with intended users and taking into account audience factors such as culture, race, ethnicity, language, access, functional needs (ie, disabilities), and expectations [35,36]—are likely to increase clinicians’ knowledge, perceptions, attitudes, and practice behaviors [37-42]. Numerous Web-based HPV vaccine educational interventions have been introduced to accommodate clinicians’ educational needs [43]. However, an extensive and comprehensive review (of CINAHL, MEDLINE, ERIC, and Ebsco Academic Complete databases, using variations and Boolean connectors with the following terms: “online intervention,” “online program,” “HPV vaccine,” “clinicians,” “physicians,” “nurses and evaluation”) demonstrated that there has been no rigorous evaluation of the design, content, and usability levels of these programs. Without such evaluation data, it is unknown whether such interventions are achieving their intended outcomes, and which aspects of the interventions should be refined [44,45].

Evaluation of the leading Web-based HPV vaccine educational interventions is important in guiding efforts to promote clinician recommendations for the HPV vaccine [44,46]. Evaluation of Web-based interventions, using established health communication and education design principles can (1) identify strengths and weaknesses of educational interventions from the users’ perspective; (2) determine whether interventions are worth the time, resources, and expense for continued implementation; and (3) provide the evidence for designing optimally effective interventions [47]. Additionally, evaluation data can provide insights into any negative unintended consequences of the interventions, such as boomerang and iatrogenic effects [48,49] and ensures that interventions address audiences’ unique needs, culture, and expectations [35]. Evaluation research is vital not only to improve health outcomes but also to ensure that interventions are cost-effective [50]. Estimated health care cost in the United States was $2.7 trillion (18% of the gross domestic product) in 2011 [51], leading policy makers to prioritize identifying effective, evidence-based methods to prevent disease and manage rising health costs [50].

Rigorous evaluation is a central component of developing successful health education interventions [45,52] and essential for understanding clinicians’ educational needs and assessing outcomes [50]. However, current Web-based HPV vaccine interventions developed for clinicians have not been evaluated using health education and design principles. Therefore, the objectives of the study were to: (1) systematically identify HPV vaccine Web-based educational interventions developed for clinicians, and (2) evaluate the quality of Web-based educational interventions as defined by access, content, design, evaluation, interactivity, and use of theory or models to create the interventions.

## Methods

### Sample

We systematically identified current HPV vaccine Web-based educational interventions by examining general search engines (eg, Google), continuing medical education search engines (eg, PedsUniversity, MedScape), health department websites, and professional organization websites. The following search terms and variations of search terms were used within each of the search engines and websites: “clinicians,” “healthcare providers,” “HPV webinars,” “HPV vaccination webinars,” “HPV online education,” and “HPV continuing medical education.” Web-based educational interventions were included if they were (1) created for clinicians (defined as individuals qualified to deliver health care services, such as physicians, clinical nurses, and school nurses, to patients aged between 9 and 26 years); (2) delivered information about the HPV vaccine and how to increase vaccination rates; and (3) provided continuing education credits. Interventions were excluded if they were created for patients, parents, or adolescents, or if they focused on vaccines other than the HPV vaccine. We conducted the search from April 2016 to August 2017 and identified a total of 178 interventions. Of these, 21 interventions met all 3 research criteria for this study.

### Instrument

The study team developed a quality evaluation framework based on criteria established in the literature for evaluating health-related websites [53-56]. The quality evaluation framework assessed Web-based interventions using 6 key indicators: access, content, design, evaluation, interactivity, and theory or models [53-56]. Each key indicator was scored using various subindicators: higher scores for the indicators designated higher quality interventions.

Internal reliability of the subindicators was calculated using the Krippendorff’s alpha coefficient (K-alpha; for additional information please see De Swert, 2012) [57]. This coefficient was selected because it provides information on the reliability of variables, not coders, and its robust calculations are not impacted by sample size, multiple coders, or missing data [58]. After calculating Krippendorff’s alpha coefficient for 2 rounds of samples with 2 independent coders (Dr Rosen, a PhD trainer faculty member with expertise in HPV vaccination uptake, and a doctoral graduate research assistant in health education), the evaluation tool was considered to be internally reliable given that all indicator scores were above .80, which is considered the norm for acceptable reliability [57].

#### Access

To examine access of the educational interventions [53,54], 2 subindicators were used to measure different components of access. These subindicators included whether registration was required to access the intervention (score ranging from 0-1) and the cost of the intervention (score ranging from 0-1).

#### Content

Content was evaluated using 7 subindicators [53,55]: identification of purpose (score ranging from 0-1), date on which the information was updated (score ranging from 0-1), presentation of clear references (score ranging from 0-1), and links to other sources (score ranging from 0-1). Additionally, reliable sources (score ranging from 0-1) were assessed and whether the intervention included reliable sources, the type of source (eg, Centers for Disease Control and Prevention, National Institutes of Health, published peer-reviewed literature) was included in the scoring metric. The final 2 subindicators included a statement indicating that content was developed or reviewed by experts (score ranging from 0-1) and a statement of disclosure of authors, sponsors, or developers (score ranging from 0-1).

#### Design

The design components of the interventions were evaluated by layout and graphics [53]. The layout of the intervention was assessed by examining font and line spacing. Specifically, font was assessed by whether the style was easy to read (score ranging from 0-1), size was easy to read (score ranging from 0-1), text color and page color contrast were easy to read (score ranging from 0-1), and line spacing was easy to read (score ranging from 0-1). Graphics were assessed to determine if they were clearly labeled, and scores ranged from 0 to 3 with 0 indicating 0% of graphics were labeled, 1 indicating a minimum of 25% of the graphics were labeled, 2 indicating a minimum of 50% of the graphics were labeled, and 3 indicating a minimum of 75% of the graphics were labeled.

#### Evaluation

Evaluation was assessed using 3 subindicators [53-55]: whether participant outcomes were evaluated (eg, knowledge and attitudes; score ranging from 0-1), the level of that evaluation (score ranging from 0-2; 0 indicating no evaluation, 1 indicating an evaluation of HPV or HPV vaccine knowledge, and 2 indicating an evaluation of HPV or HPV vaccine attitudes) and whether the participant was provided an opportunity to evaluate the intervention (score ranging from 0-1).

#### Interactivity

The indicator for interactivity included 2 subindicators [54,55]. The first subindicator assessed whether there was a location for participants to direct questions during the educational intervention (score ranging from 0-1). The second subindicator assessed whether the intervention included any interactive components (score ranging from 0-1). If the intervention included any interactive component, the interactive component was recorded in the scoring metric. The interactive components included discussion boards, “ask the expert” bulletin boards, sign up for email reminders, sign up for newsletters, and other interactive components.

#### Theory and Models

The theory and models indicator was assessed by examining whether there was an explicit statement that a theory or model was used to develop the intervention (score ranging from 0-1) [54-56]. If a theory or model was used to develop the intervention, the theory or model was recorded in the scoring metric.

### Procedure

Once interrater reliability was established for the evaluation tool with all indicator scores above .80, 2 independent coders (Mr Bishop and Ms McDonald) utilized the tool to evaluate the educational interventions identified. One of the coders, Mr Bishop is a health education doctoral student with expertise in sexuality education and evaluated the first 11 interventions. The other coder, Ms McDonald is a health education doctoral student with expertise in school health and evaluated the remaining 10 interventions. Frequency distributions were calculated for each of the subindicators to determine specific strength and weaknesses of the interventions. Because this study assessed access, content, and design aspects of interventions and did not include human subjects; this study is considered nonhuman subjects research and hence institutional review board approval was not required.

## Results

### Intervention Characteristics

A total of 21 interventions were identified out of which, 7 (33%) were webinars; 7 (33%) documentary, TV series, or videos; and 7 (33%) other (eg, text article, modules). Medscape, a health information website, provided 10 (48%) interventions, Continuing Nursing Education University provided 2 (10%), CDC provided 3 (14%), professional organizations (eg, American Academy of Pediatrics and Texas Medical Association) provided 3 (14%), nonprofit organizations (eg, Indiana Immunization Coalition) provided 1 (5%), a federally-authorized regional organization (The Suwannee River Area Health Education Center) provided 1 (5%), and a university (Boston University School of Medicine Continuing Medical Education and Continuing Nursing Education) provided 1 (5%). Multimedia Appendix 1 includes the characteristics of the interventions.

### Quality Evaluation

On the basis of the evaluation indicators, 13 (62%) interventions required registration, but all interventions were accessible without cost to the participant (K-alpha=1.0). Additionally, 17 (81%) educational interventions identified the purpose of the intervention (K-alpha=1.0), and 12 (57%) provided a date when the information had been updated: 7 (33%) were updated in the last 6 months (K-alpha=1.0). In assessing presentation of clear references, 18 (86%) interventions provided references (K-alpha=1.0), and 8 (38%) provided links to other sources (K-alpha=1.0). Most interventions (18/21, 85%) provided reliable references or sources (K-alpha=1.0). The references or sources included information from the CDC (n=16), published peer-reviewed literature (n=16), American Cancer Society (n=5), National Cancer Institute (n=4), Institutes of Medicine (n=4), WHO (n=2), and American Academy of Pediatrics (n=1). Of the 21 interventions, 14 (67%) had a statement of disclosures from the authors, sponsors, or developers (K-alpha=1.0).

For the design subindicators, 2 interventions were documentary or videos that did not include text, and therefore, were not included in the total sample for these subindicators. All interventions (n=19) included easy-to-read font size, font style, color, and line spacing (K-alpha=1.0 for these 3 subindicators). For the subindicator “Graphics were clearly labeled,” only 13 interventions included graphics; thus, the sample for this subindicator is 13 interventions. Out of the 13 interventions, there were 10 (77%) interventions with a minimum of 75% of graphics labeled, 2 (15%) with a minimum of 50% of graphics labeled, and 1 (8%) intervention with a minimum of 25% of graphics labeled. No intervention had 0% of graphics labeled (K-alpha=1.0).

Of the 21 interventions, 17 (81%) included an evaluation for participant outcomes: 17 (81%) assessed HPV and HPV vaccine knowledge, and none assessed attitudes toward HPV and the HPV vaccine. Furthermore, 14 (67%) interventions provided the participants the opportunity to evaluate or provide feedback (K-alpha=1.0). Five (24%) interventions included an interactive component (K-alpha=1.0). The most commonly used interactive component was a polling or knowledge check activity (n=4). No educational intervention explicitly stated a theory or model that was used to develop the intervention. Table 1 provides additional results from the evaluation, and Table 2 provides the quality summary score for each Web-based intervention.

**Table 1:** Web-based educational intervention quality evaluation results (n=21).

Indicator and subindicator		Scoring frequency	
		Yes	No
**Access**			
	Registration required	13	8
	Cost	0	21
**Content**			
	Date information was updated	12	9
	Identification of purpose	17	4
	Presentation of clear references	18	3
	Links to other sources	8	13
	Reliable references and sources^a^	18	3
	Statement indicating content was developed or reviewed by experts	20	14
	Disclosure of authors, sponsors, or developers	14	7
**Design**			
	Font style was easy to read^b^	19	0
	Font size was easy to read^b^	19	0
	Font color and page color contrast was easy to read^b^	19	0
	Line spacing was easy to read^b^	19	0
	Graphics were clearly labeled^c^	13	0
**Evaluation**			
	Evaluation for participant outcomes^d^	17	4
	Participant provided opportunity to evaluate intervention	14	7
**Interactivity**			
	Location to direct participant questions	6	15
	Included interactive component^e^	5	16
**Theory or model(s)**			
	Theory or model was used to develop intervention	0	21

^a^The Centers for Disease Control and Prevention (n=16) and published peer reviewed literature (n=16) were the most common frequency cited sources, followed by American Cancer Society (n=5), National Institutes of Health (n=4), Institute of Medicine (n=4), World Health Organization (n=2), Food and Drug Administration (n=1), and the American Academy of Pediatrics (n=1).

^b^Two interventions were a documentary or TV series that did not include any type of font or graphics for informational purposes. Therefore, for the Design subindicators font style, font size, font color, and line spacing, the sample size was n=19.

^c^For the graphic subindicator, eight interventions did not include graphics for informational purpose. Therefore, the sample size was n=13. There were 10 interventions with a minimum of 75% of graphics labeled, 2 interventions with a minimum of 50% of graphics labeled, 1 intervention with a minimum of 25% of graphics labeled.

^d^Specific levels of evaluation for participant outcomes include 17 interventions assessing HPV and HPV vaccine knowledge, and no intervention assessing attitudes toward HPV and the HPV vaccine.

^e^Five interventions provided participant interactivity. Four interventions included an interactive knowledge check, and 1 intervention included directions to email reminders and newsletters.

**Table 2:** Quality summary scores for Web-based interventions.

Intervention title^a^	Summary score (out of 25)
HPV Vaccine Safety and Efficacy	20
HPV Vaccines: Updates and Clinical Perspective	20
Increasing Adolescent Immunization Coverage	20
Don’t Wait Vaccinate! The Prevention of HPV Cancers (Part 2)	19
HPV Vaccination is Cancer Prevention (2017 Update)	19
Overcoming Gender and Socioeconomic Disparities in HPV Vaccination	19
You are the Key to HPV Cancer Prevention	18^b^
Don’t Wait Vaccinate! The Prevention of HPV Cancers	17
Immunization: You Call the Shots-Module Eight-HPV, 2016	17
Immunization: You Call the Shots-Module Eighteen—Vaccine Administration	17^c^
You are the Key to HPV Cancer Prevention	17^d^
ACIP Releases Pediatric Vaccine Schedule	16^c^
Adolescent Immunizations: Strongly Recommending the HPV Vaccine	16
AAP Provides Guidance for Parents Who Refuse Vaccination	15^c^
ACIP Releases Adult Vaccine Recommendations	15^c^
CDC Updates Guideline Recommendations for HPV Vaccination	15^c^
Human Papillomavirus (HPV) Vaccine Safety	15^c^
The Story of HPV: Yesterday, Today, and Tomorrow	14
HPV Vaccines: Updates and Clinical Perspective	13
Putting HPV Vaccine Knowledge Into Practice	7^e^
HPV Documentary—Division of Continuing Medical Education	2^e^

^a^HPV: Human Papillomavirus; ACIP: Advisory Committee on Immunization Practices; AAP: American Academy of Pediatrics; CDC: Centers for Disease Control and Prevention.

^b^You are the key to HPV Cancer Prevention intervention published 9/2/2015 and expires 9/7/2017.

^c^These interventions did not include any type of graphics for informational purpose. Therefore, the total score is out of 24.

^d^You are the key to HPV Cancer Prevention intervention published 4/21/2016 and expires 4/21/2018.

^e^These interventions were documentaries and did not include any type of font or graphics for informational purposes. Therefore, the total score is out of 20.

## Discussion

### Principal Findings

This study provides a systematic, evidence-based assessment of the strengths and weaknesses of current HPV vaccine Web-based educational interventions. Strengths of the assessed Web-based educational interventions include: (1) being developed by experts in the field; (2) providing reliable references or sources; (3) providing clinicians with access to CEs for no cost; (4) following basic design principles with easy-to-read fonts, colors, and graphics; and (5) consistently providing evaluation opportunities for participant knowledge outcomes. Weaknesses of the educational interventions included lack of: (1) evaluation of outcomes including participants’ attitudes about HPV vaccination, intention to recommended vaccination, and recommendation of behaviors; (2) theory-based interventions; (3) opportunity for participants to provide feedback or evaluation of the intervention; (4) links to other sources or resources; and (5) interactivity throughout the intervention.

HPV vaccination rates are well below the Healthy People 2020 objective [8], and clinicians report that they would benefit from CE regarding the HPV vaccine [29]. Because clinicians’ HPV vaccine recommendation is one of the most important predictors of HPV vaccination uptake [59-62], ensuring that clinicians are equipped with current and accurate information is critical [63]. Clinicians, however, are continually challenged in providing parents and patients with evidence-based HPV vaccine information because of changing vaccine guidelines and the volume of information and sources available [64]. Thus, clinicians’ report obtaining a large portion of HPV vaccine information from professional organizations [65] possibly because of lack of time needed to identify multiple sources of accurate information [66]. In this study, only 3 interventions were provided by 2 professional organizations, including the American Academy of Pediatrics and the Texas Medical Association. Medscape, a health information website, provided almost half of the interventions. Given that professional organizations are cited by clinicians as an important and trusted source of HPV vaccine information, professional organizations need to increase efforts to collaborate with health information websites and other organizations and institutions to provide evidence- and theory-based interventions. A recent study demonstrated that organizations working on cancer research identified the ability to leverage resources, lower costs, increase organization reputation, and the development of new tools and methodology as benefits to interorganizational collaboration [67]. Therefore, interorganizational collaboration to provide clinicians with HPV vaccine Web-based interventions has the potential to improve outcomes related to HPV vaccination rates and cancer risk reduction.

We found that none of the interventions examined included a statement that a theory was used to create the intervention. To improve outcome behaviors and increase clinician HPV vaccine recommendation behaviors, intervention developers should utilize science and evidence that supports effective medical education and behavior change [54]. Theories can be used for quality assessment and improvement by identifying factors contributing to behavior change and which factors are ineffective. Overall, interventions based in theory provide an advantage in changing behavior by providing a logical and systematic approach to increasing clinicians’ recommendation of the HPV vaccine [54].

None of the Web-based educational interventions included in this study evaluated HPV attitudes, intention to recommend vaccination, or actual recommendation behavior. This is concerning given that clinician attitudes are an important predictor of vaccine recommendations. Clinicians have reported concerns regarding HPV vaccine safety [68-70], a lack of self-confidence in providing strong vaccine recommendations [15,27,28], and belief that it is not important for adolescents to receive the HPV vaccine at the recommended age of 11 to 12 years [71]. Therefore, interventions should be designed with the goal of changing clinician attitudes and vaccine recommendations, and evaluation of these outcomes is a key component of successful interventions [45,52]. Evaluation of outcomes is also important for the translation of health communication research into efforts to promote clinician recommendations of the HPV vaccine [44,46]. Finally, evaluation is essential for understanding clinicians’ educational needs and assessing program outcomes addressing important health issues [50].

Although face-to-face educational interventions have shown to improve clinicians’ willingness to provide immunizations and routinely screen immunization records at visits [72], evaluations specifically assessing HPV-related Web-based educational interventions are limited [73]. Only 2 published studies provided evaluation results on webinars designed to increase adolescent vaccination rates. Results suggest webinars have the potential to increase clinician recommendation behaviors and adolescent Tdap, meningococcal, and HPV vaccination rates similar to in-person educational interventions [74,75]. Web-based educational interventions create a unique platform to provide clinicians with the knowledge and skills needed to promote the HPV vaccine among adolescents. One important component of Web-based educational interventions is interactivity [54,55]. Interactive components encourage users to be actively involved in the intervention and have been linked to short-term behavioral improvements [76]. Furthermore, Kreps and Neuhauser pinpoint interactivity as a communication attribute with the ability to exponentially improve health promotion [76]. Even though interactivity can have a significant impact on participants, only 5 interventions from this study included an interactive component. Because Web-based educational interventions continue to gain popularity because of convenience and economic benefits [31], the lack of interactivity in the majority of HPV-related Web-based educational interventions is alarming given the research supporting the importance of interactive components. More research should be conducted to determine the impact of interactive components in HPV-related Web-based educational interventions on clinicians’ HPV vaccination recommendation behaviors.

### Limitations

While this study provides innovative insight into the quality of Web-based HPV vaccine educational interventions created for clinicians, there are limitations that should be considered. First, only Web-based educational interventions were evaluated, and these results cannot be generalized to other types of interventions such as face-to-face lectures, grand rounds, or seminars. There would be substantial benefit to conducting evaluations of face-to-face lectures and seminar materials to assess all venues of continuing education for clinicians regarding the HPV vaccine. Second, this quality evaluation did not assess participants’ experience of the intervention and therefore, cannot identify every area for improvement. Data were not collected from participants themselves regarding usability: this study identified only 7 indicators of usability. Third, this study was a quality evaluation and did not evaluate participant outcomes (eg, knowledge, attitudes, recommendation behaviors) after completing the intervention. Fourth, the evaluation was conducted using only the materials that were accessible at the time of the study, and there is the potential that a component (such as, a follow-up emailed evaluation after the intervention to participants) was not included in this evaluation. Despite these limitations, these findings provide valuable information for those who develop Web-based continuing education interventions regarding HPV vaccines, by providing a quantitative approach to identifying the design and usability strengths and weaknesses of HPV vaccine Web-based educational interventions.

### Future Work

The data resulting from this study have the potential to help shift current research practice paradigms. The findings suggest that those who develop Web-based educational interventions to promote HPV vaccine recommendations utilize design science principles, a powerful approach and process that includes participatory action research to iteratively develop and evaluate health education interventions [77]. Additional qualitative, multi-approach evaluation research is needed to further assess the content (eg, the specific messages provided to clinicians about the HPV vaccine and recommendation behaviors) and usability of these interventions from the participants’ perspective. Further evaluation research is needed to ensure that interventions are being developed using all design principles and are effective at increasing strong and consistent HPV vaccine recommendations from clinicians.

### Conclusions

The quality evaluation of these interventions demonstrated that Web-based interventions were based on reliable sources, developed by experts, and were created with critical design aspects (eg, font style, size, and color were easy to read, graphics were clearly labeled). However, there were limited outcome evaluations for users measuring attitudes, intentions, or behaviors, and lack of user interactivity. Results from this study suggest best practices for designing, refining, and implementing Web-based interventions to promote HPV vaccination within the clinician population.

## Appendix

Multimedia Appendix 1 Intervention characteristics.

## Abbreviations

AAP: American Academy of Pediatrics
ACIP: Advisory Committee on Immunization Practices
CDC: Centers for Disease Control and Prevention
CE: continuing education
HPV: human papillomavirus
