# Supplementary material for: Quality of Web-Based Educational Interventions for Clinicians on Human Papillomavirus Vaccine: Content and Usability Assessment
Source: JMIR Cancer. 2018 Feb 16;4(1):e3. doi: 10.2196/cancer.9114 (PMC5834755; doi:10.2196/cancer.9114)
Supplement: Multimedia Appendix 1 [file cancer_v4i1e3_app1.pdf]

Multimedia Appendix 1**.** Intervention characteristics.

				Intervention Type		
Intervention	Organization	Date updated	Expiration date	Webinar	Documentary or TV series	Other (eg,, text article, module)
You Are the Key to HPV Cancer Prevention	CDC	3/23/2017	4/21/2018	●		
Adolescent Immunizations: Strongly Recommending the HPV Vaccine	American Academy of Pediatrics	10/8/2014	10/8/2017	●		
HPV Documentary	Indiana Immunization Coalition	7/1/2015	2018		●	
You are the Key to HPV Cancer Prevention	Suwannee River Area Health Education Center	9/7/2015	9/7/2017	●		

Immunization: You Call the Shots – Module Eight – HPV, 2016	CDC	2/17/2016	1/27/2018			●
Don’t Wait – Vaccinate! The Prevention of HPV Cancers	Texas Medical Association	12/31/2015	12/31/2018	●		
Don’t Wait – Vaccinate! The Prevention of HPV Cancers. Part 2	Texas Medical Association	1/21/2016	1/21/2019	●		
Human Papillomavirus (HPV) Vaccine Safety	CME University	5/10/2017	5/10/2018			●

HPV Vaccines: Updates and Clinical Perspective	Medscape	2/28/2017	3/24/2018		●	
The Story of HPV: Yesterday, Today, and Tomorrow	Medscape	3/24/2017	3/24/2018		●	
Putting HPV Vaccine Knowledge Into Practice	Medscape	6/14/2017	6/14/2018		●	
CDC Updates Guideline Recommendations for HPV Vaccination	Medscape	1/26/2017	1/26/2018			●

HPV Vaccine Safety and Efficacy	Medscape	5/22/2017	5/22/2018		●	
Overcoming Gender and Socioeconomic Disparities in HPV Vaccination	Medscape	3/29/2017	3/29/2018		●	
ACIP Releases Pediatric Vaccine Schedule	Medscape	3/23/2017	3/23/2018			●
AAP Provides Guidance for Parents Who Refuse Vaccination	Medscape	9/29/2016	9/29/2017			●

ACIP Releases Adult Vaccine Recommendations	Medscape	3/16/2017	3/16/2018			●
HPV Vaccines: Updates and Clinical Perspective	Medscape	2/28/2017	2/28/2018		●	
HPV Vaccination is Cancer Prevention	Boston University School of Medicine	6/15/2017	6/15/2018	●		
Increasing Adolescent Immunization Coverage	CME University	4/4/2017	4/4/2018	●		
Immunization: You Call the Shots – Module	CDC	2/22/2016	2/22/2018			●

Eighteen – Vaccine Administration						

• indicates the type of intervention: webinar, documentary or TV series, or other.
